# Of Pandemics and Zombies: The Influence of Prior Concepts on COVID-19 Pandemic-Related Behaviors

**DOI:** 10.3390/ijerph18105207

**Published:** 2021-05-14

**Authors:** Jessecae K. Marsh, Nick D. Ungson, Dominic J. Packer

**Affiliations:** 1 Department of Psychology, Lehigh University, Bethlehem, PA 18018, USA; ndu213@lehigh.edu (N.D.U.); djp208@lehigh.edu (D.J.P.); 2 Department of Psychology, Albright College, Bethlehem, PA 18018, USA

**Keywords:** concepts, COVID-19, emergent disease, health decision-making, health behaviors

## Abstract

We use a concepts and categories research perspective to explore how prior conceptual knowledge influences thinking about a novel disease, namely COVID-19. We collected measures of how similar people thought COVID-19 was to several existing concepts that may have served as other possible comparison points for the pandemic. We also collected participants’ self-reported engagement in pandemic-related behaviors. We found that thinking the COVID-19 pandemic was similar to other serious disease outbreaks predicted greater social distancing and mask-wearing, whereas likening COVID-19 to the seasonal flu predicted engaging in significantly fewer of these behaviors. Thinking of COVID-19 as similar to zombie apocalypse scenarios or moments of major societal upheaval predicted stocking-up behaviors, but not disease mitigation behaviors. These early category comparisons influenced behaviors over a six-month span of longitudinal data collection. Our findings suggest that early conceptual comparisons track with emergent disease categories over time and influence the behaviors people engage in related to the disease. Our research illustrates how early concept formation influences behaviors over time, and suggests ways for public health experts to communicate with the public about emergent diseases.

## 1. Introduction

The world watched as, in 2020, a novel disease emerged: Coronavirus disease 2019 (COVID-19). From initial descriptions as an atypical form of pneumonia [1], to comparisons to the seasonal flu [2], to direct comparisons to the 1918 flu pandemic [3], multiple ways of thinking about COVID-19 have been advanced and have evolved over the course of the pandemic. How do these comparisons influence people’s thinking about a novel disease like COVID-19 and in turn influence their pandemic-related behaviors? In this research, we examine how people might gain an understanding of a novel disease from a concepts and categories perspective. In the following, we first briefly review how concepts are used in human cognition. Then, we outline influences on the formation of new concepts, as would be necessary for people trying to make sense of a novel disease. We then present a study that examines how people categorized COVID-19 early on in the pandemic and how the concepts they employed predicted COVID-19 related behaviors over time.

### 1.1. Research on Human Concepts

Human concepts are the storage ground for knowledge of categories in the world [4,5]. People easily form concepts informed by their interactions with category members [6,7], as well as the previous knowledge they hold [8,9]. Once a person has formed a concept for a category, this concept guides behavior [10,11]. Concepts allow people to make inferences about a new entity from their knowledge of the category it belongs to, and suggest ways to behave toward that new entity [12,13,14,15]. For example, how a person categorizes an ambiguous brown blob in their backyard (e.g., is it a groundhog or a bear cub?) dictates what they expect that blob to do next and what behaviors they should engage in (e.g., go into the backyard to investigate versus definitely stay inside and watch through a window). In this way, how we categorize objects and events in the world guides how we react to them.

Importantly, categorization is not a veridical process of matching an entity to its one true category membership. Instead, a given entity in the world can often be classified in many different ways. Categories exist in hierarchies, such that a particular instance in the world (e.g., a bird) can be categorized anywhere from a very specific, narrow category (e.g., Rufous-collared Robin) to a very general, broad category level (e.g., living thing) [16,17]. Which taxonomic level is used in a hierarchy is determined by a person’s knowledge of and expertise with the category structure [18,19]. Beyond existing at multiple taxonomic levels, entities can simultaneously belong in other types of categories, such as script-based categories. For example, bacon is not just in the taxonomic category of “meat,” it also is in the script categories of “breakfast foods” and “delicious snack foods” [20,21]. The demands of a categorization task can automatically activate either taxonomic or script-based classification (e.g., bacon can automatically activate “meat’ or “breakfast foods”), with the category that is activated dictating the inferences that are drawn for the category member [21]. Although the previous examples illustrate cases in which categorization is clear, entities can display features that are ambiguous as to what category is the most appropriate. In such cases, people can use current hypotheses or other top-down knowledge to make a categorization, allowing for different prior knowledge to result in different categorizations [22,23,24]. Across these areas of research, an important theme emerges: how a particular entity is categorized can be flexibly determined by the demands of the categorization task, as well as the knowledge and hypotheses of the categorizer. In turn, whatever categorization is made then guides inferences about, and behaviors related to, that category.

### 1.2. Concepts in the Time of COVID-19

We have described human concepts as central to guiding behavior. This basic tenet of human concepts research becomes critically important when we try to understand how people think about emergent health categories such as COVID-19. How people think about a disease category can change the behaviors they engage in related to the disease [25,26,27]. For example, the categorization of a health disorder (e.g., mental versus physical illness; biologically versus psychologically based disease) dictates what treatment is seen as most appropriate for the disorder [28,29,30]. Similarly, beliefs about the causal structure of a health category (e.g., whether there is an underlying causal essence that creates the features of the category and is shared by all category members) can determine whether category members are stigmatized [31,32,33]. Overall, this research demonstrates that the nature of people’s concepts of a disease shapes how they respond to it, suggesting a similar influence of concepts in responding to COVID-19.

How do people form a new concept for an emergent disease such as COVID-19? A large amount of literature has explored how people learn category structures and form new concepts [34,35,36]. Learning real-world concepts is robustly influenced by prior knowledge [8,37,38,39,40]. Concepts people already hold can be used as a starting place into which they can integrate knowledge about new categories, as a lens through which to select features or attributes to attend to in learning, as a way to interpret new ambiguous information in light of the concept they hold, or just as a general facilitator of new learning (as reviewed by Heit in [41]). In thinking about learning a new concept for a novel disease such as COVID-19, people’s prior conceptual knowledge could be a starting point for forming that concept through any or all of these means. The more similar categories seem to each other, the stronger inferences people draw across those categories [14,25]. In this way, a prior concept that is seen as highly similar to COVID-19 may serve as a starting block for a new concept of COVID-19, and function as a foundation to make inferences and reason about the new disease.

The prior conceptual knowledge people use to interpret or integrate information about COVID-19 could affect the form that new concept takes, and consequently influence behavior. What concepts could have guided thinking about COVID-19 early in the pandemic? We consider a set of varied concepts that seem to be reasonable comparison points for disease, as well concepts drawn from how people were informally talking about the pandemic early in its onset.

First, people could think of COVID-19 as similar to a common disease category they have experience with, namely seasonal influenza. Early in the pandemic, epidemiological efforts to understand the spread of COVID-19 likened its transmission patterns to the seasonal flu, basing mitigation recommendations on those for the flu [42]. Perceiving this public health advice (e.g., hand washing, covering mouth while coughing) as similar to advice for the flu could have promoted people using their concepts of the seasonal flu to think about COVID-19. Additionally, political efforts by some politicians to underplay the seriousness of COVID-19 early in the pandemic by comparing it to the seasonal flu [2] could have additionally supported likening COVID-19 to the flu.

Alternatively, people may have drawn on concepts of other pandemics and epidemics to understand COVID-19. Parallels can be drawn between COVID-19 and other serious diseases, depending on what feature of the disease is focused on. For example, focusing on the global nature of COVID-19 could result in comparisons to the 1918 flu pandemic, whereas thinking about the similar viral origins could draw comparisons to other coronavirus-caused diseases such as SARS. Even focusing on the public mitigation behaviors, such as mask-wearing and quarantining, could result in thinking of COVID-19 like historically depicted diseases, such as plagues.

Finally, the far-reaching disruptions COVID-19 produced in normal daily functioning could have resulted in categorizing COVID-19 with things outside the health realm. Supplies running low in stores may have evoked ideas of apocalyptic events depicted in popular media (e.g., zombie scenarios). Seeing the shuttering of businesses and the prospect of mass unemployment may have brought to mind similar moments of major upheaval in history (e.g., the Great Depression). We thank Time 1 participants for thinking of this upheaval category.

Importantly, we predict that the prior concepts people use to guide building a concept for COVID-19 will shape their behavioral reactions to the pandemic. Just as categorizing an ambiguous backyard blob as a groundhog or a bear cub results in different judgments of the wisdom of leaving the house, how people map COVID-19 to their prior concepts should likewise guide what they think is safe and appropriate behavior in response to the pandemic (e.g., should I leave my house?). Specifically, aligning COVID-19 with other serious diseases could focus people on the mortality and contagion elements of the disease. We predict that using concepts of serious disease (like the 1918 flu or SARS) to think about the pandemic should result in people conceiving of COVID-19 as serious, causing them to engage in behaviors that are necessary to stop the spread of major contagious diseases, such as social distancing.

In contrast, thinking COVID-19 is similar to normal seasonal flu should promote less engagement in the same behaviors. People do not tend to conceptualize the seasonal flu as a major health risk, and often do not seek out available annual flu vaccinations [43,44]. Thinking of COVID-19 in a similar way to the flu should suggest that social distancing and other mitigation behaviors are relatively unimportant. Additionally, politicians likened COVID-19 to the flu with the direct goal of suggesting why social distancing and lockdown measures were not needed [2]. We predict that conceptualizing COVID-19 like the seasonal flu should result in people showing a lower rate of engaging in mitigation behaviors.

Finally, if people are using prior concepts of apocalyptic or large-scale upheaval events to understand the pandemic, we speculated that they might be attentive to aspects of the pandemic other than just the health-related implications, including disruptions to daily life and, in particular, potential shortages of important goods (e.g., food and toilet paper). Thus, we predict that people using such concepts would engage in behaviors that prepare for future shortages by stocking up on supplies.

In exploring how prior concepts guide reactions to COVID-19, we can also examine how this influence on behavior may change over time. It is possible to make contrasting predictions about this influence. As people gain more experience with a category, their concepts of that category change and refine [9,45,46]. For example, experts’ concepts within their domain of expertise are more detailed and represent different knowledge than laypeople’s concepts in the same domain [18,19,47,48,49,50,51]. We could expect people’s concept of COVID-19 to evolve over the course of the pandemic. If this is the case, early comparison categories for COVID-19 may influence behavior less over time, as a unique concept for COVID-19 is built that grows more distinct from other held concepts.

Alternatively, initial categorizations of an instance can guide how people interpret new information for categorization that they encounter over time [8,23,24,52,53]. Likewise, the first categorization given a novel exemplar can stick with the entity and guide expectations even when new categorizations are suggested [54]. From this research we could predict that while a person’s concept of COVID-19 may have changed over time, incoming knowledge about the pandemic was fundamentally shaped by prior knowledge, permanently aligning the new concept with the concepts used for comparison. Overall, this would predict that early categorizations may influence behaviors in similar ways over time. By examining the influence of concepts applied early in the pandemic over time, we can develop a better understanding of whether early conceptual alignments permanently direct the course of how novel diseases are perceived.

### 1.3. The Current Experiment

To test our predictions, we present a study that explored how likening COVID-19 to different prior concepts influenced people’s pandemic response behaviors over six months of the pandemic. We use data collected as part of a larger longitudinal study that included a battery of questions related to perceptions of COVID-19. For the research question examined in this paper, we collected data on how similar people thought COVID-19 was to other categories of disease, as well as to categories not related to health. We also collected a series of COVID response behaviors, including social distancing, mask-wearing, vaccination uptake willingness, and stocking up behaviors. Our design allows us to test whether categorizations early in the pandemic influence engagement in these behaviors over the course of the pandemic.

## 2. Methods

We explored through online surveys how aligning COVID-19 with previously held concepts of serious disease, normal flu, apocalypse, or upheaval predicted pandemic-related behaviors across four time points. We specifically explored behaviors geared at mitigating the disease (i.e., social distancing, mask-wearing, and vaccination), as well as behaviors related to non-health aspects of the pandemic (i.e., stocking up behaviors). We predicted that prior concepts used early in the pandemic will guide behaviors making people more or less likely to engage in mitigation and stocking up behaviors. We additionally explored whether these influences hold over time.

We used variables collected as part of our larger study to see if our predictions held above and beyond other published predictors of adopting mitigation behaviors. Given the politicization of many mitigation behaviors in the United States, as well as partisan comparisons of COVID-19 to the seasonal flu, we included political orientation in our analysis as a control variable [55]. Additionally, we included the degree to which respondents perceived themselves to be at heightened risk from COVID-19 as a control variable, given the role of risk perceptions in predicting disease response behaviors in general [56,57] and for COVID-19 specifically [58]. Including these variables allowed us to investigate the impact of categorization above and beyond other key variables that have been shown to guide COVID-19 responses.

We posted our surveys on four separate dates, with data collection running until no new data were incoming (a span of 1 to 4 days, depending on time point). The first two times represent early points in the COVID-19 pandemic when quarantine orders were new in communities (Time 1 (T1): 6 April 2020; Time 2 (T2): 21 April 2020). We collected a third time point in the summer when people had experienced social distancing and other mitigation efforts for several months (Time 3 (T3): 5 June 2020). We collected a fourth time point in the fall when cases were again increasing nationally (Time 4 (T4): 1 October 2020). Analyses using these same data collection points but examining other variables from the larger set or discussing other parts of the larger project can be found in other publications [59,60]. Copies of our surveys, data, and code used to analyze our data can be found here: https://osf.io/b4sn9 (accessed on 9 April 2021).

### 2.1. Participants

All participants were recruited using the Prolific online platform (Prolific.co). Prolific is an online resource for recruiting people interested in participating in online research studies. Participants (*N* = 2506) were invited at T1 and then recontacted at each subsequent time point to participate. We analyzed the set of 1773 participants who completed our main measures of interest for these analyses (i.e., category similarity questions and behavioral outcomes) at all four time points. Participants in this set of analyses predominantly identified as white (*n* = 1207) and female (*n* = 938). The mean age was 33.56 (age range 18–78). Our participants were geographically dispersed throughout the United States and liberal-leaning in their politics (*M* = 3.97, *SD* = 2.40 on a scale from 1 (*very liberal*) to 10 (*very conservative*)).

### 2.2. Materials

#### 2.2.1. Category Similarity Measures

Our goal was to develop items that represented categories people would have prior knowledge of that could be relevant to the formation of a COVID-19 concept. To do this, we developed category items via data collection at T1 and T2. As an overview, we created items at T1 that fell into three broad comparison categories that a priori we thought could serve as reasonable prior concepts for thinking about COVID-19: serious disease in the form of previous pandemics, the seasonal flu, and apocalyptic events in the form of zombie media. We additionally allowed participants at T1 to generate free responses of other possible category comparisons. After inspecting the T1 free response data, we included new categories at T2 that emerged from those data. From these two rounds of testing, we created a final set that mapped to four categories: **Serious Diseases**, **Normal Flu**, **Apocalyptic Events**, and **Major Upheavals**. We additionally conducted a factor analysis to determine if our grouping of items fit the structure of our data. The factor analysis broadly confirmed our a priori category assignments. The final set of items that were used for calculating our category similarity measures are found in Table 1. Further details on the selection of our category items can be found in Appendix A.

To measure how our targeted comparison categories were being applied to COVID-19, we asked participants to make a similarity judgment with the prompt, “For each of the items below, please indicate how similar you think each event or example is to the current spread of COVID-19”, on a scale of 0 (*not at all*) to 10 (*extremely similar*). The slider scale we used for these judgments showed participants the number they chose. Due to a coding error, the scale visually displayed labels for every value from 0 to 10 except for the number 5 (the scale label of 4 was followed by the label of 6). This was true for all similarity ratings across T1 and T2. Participants could still respond with any number between 0 and 10, and across participants all scale values (including 5) were used for all items.

#### 2.2.2. COVID-19 Behavioral Response Measures

We measured a series of COVID-19 behavioral reactions by asking participants to report “what actions you personally have taken in response to COVID-19” on a scale of *Not at all*, *Occasionally*, *Frequently*, and *All the time*. For these analyses, we focus on four major reactions to COVID-19. First, at T1 through T4, we had participants report the extent to which they were engaging in seven **social distancing** behaviors (i.e., maintaining physical distance from others, staying home as much as possible, limiting trips to stores, avoiding restaurants and other public places, canceling social engagements, avoiding family gatherings, and changing travel plans; 0.81 < αs < 0.86), as well as four **stocking up** behaviors (i.e., stocking up on household supplies such as toilet paper and toothpaste; food; sanitizer and disinfectant; protective equipment, including face masks; 0.83 < αs < 0.89). By T3, face masks had become a growing practice and eventual recommendation. As such, at T3 and T4, we additionally asked participants to report **mask-wearing** (i.e., wearing a face mask in public places). Finally, at T4, when the research on vaccines had greatly progressed, we measured **vaccine willingness** with the following question: “If and when a clinically tested vaccine for COVID-19 becomes available, how quickly would you be willing to take it?”. Participants chose a response from a six-point scale: *immediately, after a month, after a few months, after a year, after two or more years,* and *I will never be willing to take a COVID-19 vaccine.* When analyzing vaccine willingness, we always excluded participants who indicated they would “never be willing” to take the vaccine, thus treating the remaining choice options as a continuous scale from 1 (*immediately*) to 5 (*after two more years*).

#### 2.2.3. Controls for COVID-19 Behavioral Response Measures

In all analyses, we also controlled for two known predictors of COVID-19 behavioral responses. First, we included one item that measured political orientation: “How would you rate your political outlook or worldview?” on a scale of 1 (*very liberal*) to 10 (*very conservative*). We also controlled for perceived self-risk, which was measured using two items: “Do you see yourself as being vulnerable to the more severe impacts of COVID-19? In other words, do you see yourself as having a higher likelihood of hospitalization than the majority of the population?” and “Do you see yourself as someone who is in an at-risk population for COVID-19?”. Participants responded to the self-risk items by selecting “Yes,” “No,” or “Unsure”. Participants who endorsed “Yes” to either or both of these questions were coded as perceiving high self-risk (*n* = 508); all others were coded as perceiving low self-risk (*n* = 1259).

### 2.3. Procedure

At each time point, participants began the study by completing an informed consent document. Participants then completed a series of questions that asked about their perceptions of COVID-19 along with their perceptions of their local community. Similar sets of questions were blocked together, with the order of blocks randomized. For a given participant, the category similarity items were always presented as one of the first three blocks of the study. All category similarity items were presented on the same screen. The COVID-19 behavioral response measures always came after the category similarity measures and were separated by questions on other topics not of relevance to this investigation. Demographics questions were always presented last. The order of the category similarity items and the order of behavioral response measures were randomized for each participant at every time point.

## 3. Results

### 3.1. Analysis Plan

We averaged across each participant’s category similarity ratings for items in a given category (as seen in Table 1) to create mean category similarity scores for each category. The category similarity scores were as follows for perceiving COVID-19 as similar to: Serious Diseases (*M* = 4.88, *SD* = 1.65), Normal Flu (*M* = 4.37, *SD* = 2.62), Apocalyptic Events (*M* = 2.16, *SD* = 2.00), and Major Upheavals (*M* = 3.34, *SD* = 1.91). We likewise averaged the estimates of the seven social distancing items to create a mean social distancing score, and the four stocking up behaviors to create a mean stocking up score. Mask-wearing and vaccine willingness were measured with one item at each timepoint.

We used multiple regression to analyze how ratings of category similarity collected at T1 and T2 (i.e., Serious Diseases, Apocalyptic Events, Major Upheavals, and Normal Flu) predicted self-reported pandemic-related behaviors of social distancing, stocking up, mask-wearing, and vaccine willingness at T2, T3, and T4. At each time point, for each behavior, we conducted a two-step regression. In Model 1, we regressed the behavior onto political orientation and self-perceived risk due to COVID-19 (both measured at T1). In Model 2, we added all four category ratings; this allowed us to examine relationships between category ratings and behaviors over and above the influence of political beliefs and perceived personal risk. Below, we describe how political orientation and perceived self-risk predict behaviors (i.e., Model 1). Then, we describe how each category rating uniquely predicted behaviors across time points (i.e., Model 2). See Table 2, Table 3, Table 4 and Table 5 for complete regression results from Model 2 for all behavioral outcomes.

### 3.2. Political Orientation and Perceived Self-Risk Control Measures

Political orientation consistently predicted social distancing and mask-wearing. At all time points, more conservative (compared to liberal) respondents reported engaging in less distancing and less mask-wearing (all *p*s < 0.001). Additionally, political conservatives indicated that they would wait longer to take the vaccine at T4 (*p* < 0.001). These findings replicate other published work on partisanship and COVID-19 response behaviors [55].

Perceived self-risk also consistently predicted social distancing and mask-wearing at all time points, such that respondents who saw themselves at greater risk for serious outcomes due to COVID-19 reported significantly more of these behaviors (all *p*s < 0.05), replicating published work [58]. Respondents who believed themselves at greater risk also reported more stocking up behaviors at all time points (all *p*s < 0.05).

### 3.3. Relationship between Categories and Health Behaviors

#### 3.3.1. Serious Diseases

**Figure 1:**
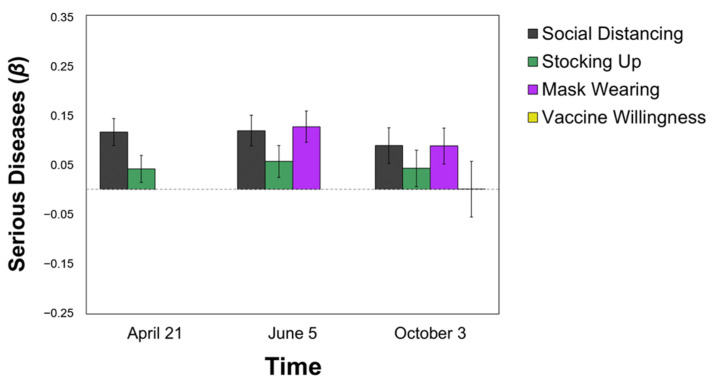
Predicting Pandemic Behaviors from the Serious Disease Categorization. All values are standardized regression coefficients (*β*) from Model 2 (i.e., over and above political ideology and perceived self-risk). Error bars represent standard error.

Perceiving COVID-19 as similar to the Serious Diseases category was positively associated with the health-protective behaviors of **more social distancing** (T2: *β* = 0.115; T3: *β* = 0.118; T4: *β* = 0.088, all *p*s < 0.05) and **more mask-wearing** (T3: *β* = 0.126; T4: *β* = 0.087, all *p*s < 0.05) at all time points, over and above political orientation and perceived self-risk. That is, respondents’ categorization of COVID-19 in early and mid-April continued to predict self-reported behaviors throughout the summer and into early October 2020. The Serious Disease category was not related to stocking up behaviors (*p*s > 0.08) or vaccine willingness, (*p* > 0.99; see Figure 1).

#### 3.3.2. Normal Flu

**Figure 2:**
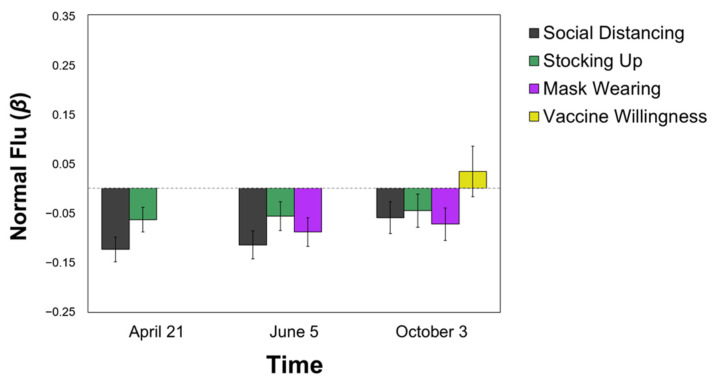
Predicting Pandemic Behaviors from the Normal Flu Categorization. All values are standardized regression coefficients (*β*) from Model 2 (i.e., over and above political ideology and perceived self-risk). Error bars represent standard error.

In stark contrast to Serious Diseases, perceiving COVID-19 as similar to the normal flu was negatively associated with health protective behaviors of **less social distancing** (T2: *β* = −0.123, T3: *β* = −0.114, *p*s < 0.001; T4: *β* = −0.059, *p* = 0.067) and **less mask-wearing** (T3: *β* = −0.088; T4: *β* = −0.072, all *p*s = 0.01) at all time points, over and above political orientation and perceived self-risk. The Normal Flu category also predicted less stocking up at T2 (*β* = −0.063, *p* < 0.05; T3 and T4 *p*s > 0.06), but was not significantly related to vaccine willingness, *p* > 0.50 (see Figure 2).

#### 3.3.3. Apocalyptic Events

**Figure 3:**
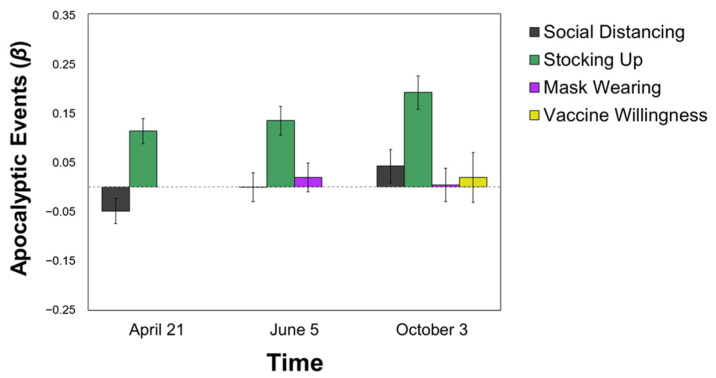
Predicting Pandemic Behaviors from the Apocalyptic Event Categorization. All values are standardized regression coefficients (*β*) from Model 2 (i.e., over and above political ideology and perceived self-risk). Error bars represent standard error.

Perceiving COVID-19 as similar to Apocalyptic Events was unrelated to health-protective behaviors of social distancing and mask-wearing at all time points. However, this category was associated with **more stocking up** (T2: *β* = 0.113; T3: *β* = 0.134; T4: *β* = 0.191, all *p*s < 0.001) at all time points, over and above political orientation and perceived self-risk. This category was also unrelated to vaccine attitudes, *p* > 0.70 (see Figure 3).

#### 3.3.4. Major Upheavals

**Figure 4:**
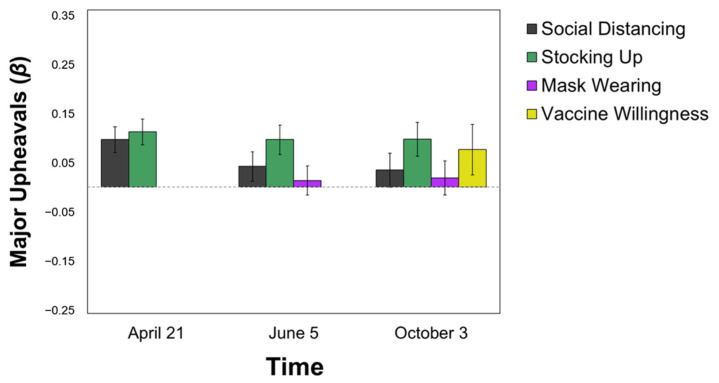
Predicting Pandemic Behaviors from the Major Upheavals Categorization. All values are standardized regression coefficients (*β*) from Model 2 (i.e., over and above political ideology and perceived self-risk). Error bars represent standard error.

Perceiving COVID-19 as similar to other types of Major Upheaval (e.g., war, natural disasters) was positively related to **more social distancing**, but only at T2 (*β* = 0.094, *p* < 0.001). It was unrelated to mask-wearing (*p*s > 0.59) and vaccine willingness (*p* > 0.15). However, similarly to the Apocalyptic Events category, this categorization predicted **more stocking up** (T2: *β* = 0.109; T3: *β* = 0.094; T4: *β* = 0.095, all *p*s < 0.01) at all time points (see Figure 4).

Taken together, we observed a reliable pattern in which comparing COVID-19 at the onset of the pandemic to a serious infectious disease, such as the 1918 flu, predicted more health-protective behaviors from late April into October. Conversely, early categorizations of COVID-19 as similar to the normal flu predicted less health-protective behaviors throughout data collection. Additionally, early comparisons of COVID-19 to an apocalyptic event or major upheaval predicted more stocking up behaviors at all time points.

## 4. Discussion

Our question of interest was how aligning COVID-19 with prior concepts early in the pandemic predicted response behaviors over the course of the pandemic. We found that likening COVID-19 to other health conditions predicted health-related behaviors over time. Namely, seeing similarity between COVID-19 and serious diseases predicted pandemic behaviors related to controlling the spread of the disease (i.e., social distancing and mask wearing), whereas likening COVID-19 with the normal flu predicted not engaging in these same behaviors. Categorizing COVID-19 as similar to non-health related categories such as apocalyptic events or major upheavals was predictive of engaging in more stocking up behaviors. All of these findings held above and beyond known predictors, such as political orientation and self-perceived risk. In this way, we present an important and novel demonstration of how finding similarity between categories can predict real-world enacted and intended behaviors.

Interestingly, we did not find that likening COVID-19 to any of our measured categories predicted willingness to receive a vaccine. A couple of issues are important to think about in interpreting this finding. One, by T4 data collection, vaccines were something that was being researched but were not available. Our participants were making a very hypothetical judgment at that time point given there was nothing widely known about the mechanism, availability, or timing of vaccine rollout. Two, thinking of COVID-19 as similar to our serious disease categories may not have predicted vaccine uptake because our serious diseases were not diseases for which a vaccine is available. For example, mapping COVID-19 to Ebola when there is no vaccine for Ebola may not inform how to think about COVID-19 vaccine willingness. At the same time, a vaccine is available for the seasonal flu, and while finding COVID-19 similar to the flu did have a positive relationship with vaccine willingness, it was not a significant predictor. This demonstrates a tension between thinking about the availability of a vaccine and its necessity. Moving forward, it will become important to understand how thinking about COVID-19 evolves as the availability of multiple vaccination options expands.

Finally, we did not include subjects who reported they were not willing to take a vaccine (*n* = 51) in our analyses of vaccine willingness in order to keep our variable continuous and because of the small number of respondents in this group. As a check, we conducted a series of exploratory logistic regression analyses to examine if comparing COVID-19 to any of our categories predicted never wanting to receive the vaccine. The only consistent predictor was political ideology, *p* < 0.001. Participants who were more conservative were more likely to say they would never receive the vaccine, over and above self-risk and any categorizations, all *p*s > 0.10. These analyses should be interpreted cautiously because of the small sample size, but suggest that our main finding that categorizations do not predict vaccine uptake is replicated in this more extreme group.

### 4.1. Implications for Research on Human Concepts

We found that relationships between prior concepts and response behaviors held, for the most part, across time points in our study. We measured category similarity at the beginning of the pandemic in April when people were settling into an understanding of the pandemic and how it impacted their lives. By our T4 data collection point in October, people were familiar with what COVID-19 was and what the pandemic entailed. Changes in conceptual knowledge could have meant that early categorizations would not matter later in our data collection as new and distinct concepts of COVID-19 were created. Instead, we found that the early categorizations of COVID-19 continued to predict behaviors across time points, up to approximately six months later.

Our findings fit with research that suggests categorizing an entity provides an interpretative lens through which information about that category member is seen [23,24,61]. In this interpretation, as new information was learned about COVID-19 (e.g., new state regulations for inside dining at restaurants), people may have interpreted it differently to support the concept they had earlier built of COVID-19. For example, if COVID-19 is thought of as a serious disease, then bans on inside dining at restaurants could be thought of as more evidence of the highly contagious nature of the disease, whereas if COVID-19 is categorized with major upheavals, the same ban may emphasize the economic situation of major upheavals. Overall, our research provides novel evidence of the persistence of early categorizations and how concepts guide real-world behavior over time.

An open question from our research is what causes people to align an emergent health disease with a particular concept in the first place. That is, how do we pick pandemics versus zombies in thinking about a new disease? The experiences people have shape the nature of their concepts [49,50,62,63]. It is possible that for COVID-19, some firsthand experiences with the disease start a path toward likening the disease to certain categories. The person who was reminded of a recently watched zombie film when they entered a grocery store and saw empty shelves may start down a different conceptual path than the person who was reminded of traveling abroad during the SARS outbreak of 2003 when seeing surgical masks worn in public. It is important to understand this process of conceptual mapping more fully in order to inform our theories of conceptual change and their implications for public health.

### 4.2. Implications for Public Health

Given the rise in emergent diseases [64,65], public health officials have to be ready to communicate information about diseases that the public knows very little or nothing about. While we tested reactions to COVID-19 specifically, we believe our results can provide guidance for public health officials more generally. Public health officials can guide how the public understands and responds to emergent diseases by how they categorize those diseases and the similarities they draw for the public. Comparing a novel disease to other serious illnesses could highlight the need to control contagion and enact mitigation behaviors.

However, it is important that public health officials pick the best disease as a comparison point to help people build their concepts. What seem like reasonable comparison choices may not always result in the desired outcomes. For example, public-facing campaigns have portrayed mental health disorders as similar to physical health conditions, promoting thinking about them as in the same category of illness. While this was done to reduce stigma, research suggests the medicalization of mental health disorders does not reduce stigma and can increase stigma for certain disorders [66,67,68]. For emergent diseases, public health officials will need to conduct research to understand what are the most useful comparisons before assuming just any serious disease will do. Once such research is done, public health officials can find the right comparison diseases for an emerging crisis, and then craft their messaging to highlight the similarity between diseases to encourage people to engage in protective behaviors.

In refining messaging, more research is needed to understand how a disease becomes understood as “serious” by the general public, and that will therefore be available to serve as a good comparison point to be used by public health officials. Our results show that likening COVID-19 to the seasonal flu predicted reduced engagement in important disease-containment behaviors. The seasonal flu is in itself a serious disease that annually kills hundreds of thousands of people worldwide [69]. Influenza variants have been responsible for recent pandemics such as the H1N1 pandemic, and are a major source for predicted future pandemics [70,71]. Despite all of this, influenza vaccination uptake in countries across the world is alarmingly lower than targeted coverage rates [72]. It is possible that future public health messaging could encourage thinking of the seasonal flu as more like COVID-19 to try to instill the seriousness of seasonal flu and encourage preventative behaviors. Further work is needed to answer this question of how people begin to take a disease more seriously.

## 5. Conclusions

Concepts guide human behavior, with reactions to COVID-19 being no exception. Our results suggest that early ways of thinking about the COVID-19 pandemic guided behaviors over the course of the pandemic. While our results sit within the specific pandemic context of COVID-19, we believe they can provide future guidance for public health officials as they seek to shape the public’s understanding of disease. In this way, understanding the best laid schemes of pandemics and zombies can help us understand how people respond to emergent disease.

## Figures and Tables

**Table 1:** Items Included in Category Similarity Scales.

Scale	Item	Time
Serious Diseases (α = 0.71)	The 1918 flu pandemic (a.k.a, the Spanish Flu)	T1
	The rise of HIV/AIDS in the 1980s	T1
	The spread of SARS in 2002–2003	T1
	Bubonic plague/black death	T2
	Swine flu/H1N1	T2
	Ebola	T2
Normal Flu	The spread of the flu in any normal year	T1
Apocalyptic Events (*α* = 0.70)	Zombie movies like *World War Z*, *I Am Legend*, or *28 Days Later*	T1
	Zombie TV shows such as *The Walking Dead*	T1
	Video and board games (e.g., *Plague Inc.*, *Pandemic*, *Fallout*)	T2
Major Upheavals (α = 0.80)	11 September 2001	T2
	Economic upheaval (e.g., the Great Depression, the 2008 recession)	T2
	Societal upheaval (e.g., the LA riots)	T2
	War (e.g., WWII)	T2

**Table 2:** Predicting Social Distancing Across Time Points Controlling for Perceived Self-Risk and Political Ideology (Model 2).

Predictor	Time 2 (β)	Time 3 (β)	Time 4 (β)
Serious Diseases	0.115 ***	0.118 ***	0.088 *
Normal Flu	−0.123 ***	−0.114 ***	−0.059
Apocalyptic Event	−0.049	<0.001	0.042
Major Upheavals	0.094 ***	0.041	0.034
Political Ideology	−0.144 ***	−0.276 ***	−0.320 ***
Perceived Self-Risk	0.056 *	0.120 ***	0.103 ***
R^2^	0.071 ***	0.134 ***	0.152 ***

*Note.* * *p* < 0.05. *** *p* < 0.001.

**Table 3:** Predicting Stocking Up Across Time Points Controlling for Perceived Self-Risk and Political Ideology (Model 2).

Predictor	Time 2 (β)	Time 3 (β)	Time 4 (β)
Serious Diseases	0.041	0.056	0.042
Normal Flu	−0.063 *	−0.056	−0.045
Apocalyptic Event	0.113 ***	0.134 ***	0.191 ***
Major Upheavals	0.109 ***	0.094 **	0.095 **
Political Ideology	0.018	−0.050	−0.057
Perceived Self-Risk	0.054 *	0.075 **	0.099 **
R^2^	0.046 ***	0.058 ***	0.086 ***

*Note.* * *p* < 0.05. ** *p* < 0.01. *** *p* < 0.001.

**Table 4:** Predicting Mask-Wearing Across Time Points Controlling for Perceived Self-Risk and Political Ideology (Model 2).

Predictor	Time 3 (β)	Time 4 (β)
Serious Diseases	0.126 ***	0.087 *
Normal Flu	−0.088 **	−0.072 *
Apocalyptic Event	0.019	0.004
Major Upheavals	0.013	0.018
Political Ideology	−0.240 ***	−0.282 ***
Perceived Self-Risk	0.119 ***	0.083 **
R^2^	0.106 ***	0.118 ***

*Note.* * *p* < 0.05. ** *p* < 0.01. *** *p* < 0.001.

**Table 5:** Predicting Vaccine Willingness at T4 Controlling for Perceived Self-Risk and Political Ideology (Model 2).

Predictor	Time 4 (β)
Serious Diseases	<0.001
Normal Flu	0.034
Apocalyptic Event	0.019
Major Upheavals	0.074
Political Ideology	0.180 ***
Perceived Self-Risk	0.046
R^2^	0.047 **

*Note.* ** *p* < 0.01. *** *p* < 0.001.

## Data Availability

The data presented in this study are available on request from the corresponding author.

## Appendix A. Creation of Category Similarity Scales

### Appendix A.1. Selecting Category Comparisons

At T1, we created items that fell into three broad comparison categories. To measure how participants likened COVID-19 to other serious diseases, items represented serious diseases of different types: (1) recent or relatively recent pandemics (3 items: 1918 flu, SARS, HIV/AIDS) and (2) fictional or religious depictions of disease (2 items: “movies about disease spread like *Outbreak*”, “the plagues described in the Bible or other religious texts”). We created a second category that represented comparison of COVID-19 to the normal seasonal flu (1 item). Finally, to measure if participants were thinking about COVID-19 in more apocalyptic terms, we developed items that fit a third category that measured if participants were categorizing COVID-19 as a generally apocalyptic event (2 items: zombie apocalypse TV shows, zombie apocalypse movies). We targeted zombie depictions of apocalypse because of their familiarity in popular culture [73]. Upon inspection of our data, we realized that our items measuring fictional or religious depictions of disease do not neatly fit into the Serious Diseases category because they include elements of disease spread, as well as apocalyptic events (e.g., Biblical plagues involve disease and destruction of communities; see factor analysis below for additional support). We reasoned that these two items may be perceived as moderately similar to both Serious Diseases and Apocalyptic Events categories. We thus dropped disease movies and religious depictions from our category scale calculations.

To complement our a priori categories, at T1 we additionally asked participants to enter free responses to the question, “Are there other historical or fictional events that you personally compare to COVID-19?”. We analyzed these open-ended data collected to see if there were any frequent or important categories that we had not included. Three of the most frequent free responses represented pandemics or widespread epidemics that we had not tested at T1: H1N1/the swine flu, Ebola, and the bubonic plague/Black Death. We included these three diseases at T2 as new potential contributors to the serious disease category. Additionally, mentioned in participants’ free responses was a new way of indexing the apocalyptic events category through an item that measured apocalyptic video and board games (e.g., the board game *Pandemic*).

In addition to exemplars related to these two existing categories, a new category emerged from the open-ended responses: moments of major upheaval. From suggestions in the open-ended responses, we included 5 new items at T2 to capture this theme of major upheaval: 11 September 2001; economic upheaval; societal upheaval; natural disasters; and war.

Finally, we re-measured two items at T2 in slightly different ways from T1 given the T1 open-ended responses. We re-measured our SARS item from T1 as MERS/SARS at T2. Additionally, people free-reported zombie books, when we had only described movies and TV shows at T1. We retested our zombie item at T2 as “zombie movies and books” to ensure that we were capturing different ways of thinking about zombies. These additions are redundant, and strongly correlated with T1 items: “MERS/SARS” (T2) with “SARS” (T1; *r* = 0.42), and “zombie movies and books” (T2) with zombie items at T1 (0.48 < *r*s < 0.49). For simplicity, we use only the T1 measures in our final category comparison items. See Table 1 for items included in final scale calculations, reliability statistics, and item wording.

All analyses presented in the main text were also conducted (1) with redundant items included, (2) with disease movies and religious depictions of disease included in the Serious Diseases category, and (3) with disease movies and religious depictions of disease included in the Apocalyptic Events category. Results were largely replicated, with 3 of 24 possible effects of category changing from marginally significant to significant, or vice versa. Importantly, none of the key conclusions of this paper are altered by these additional analyses.

### Appendix A.2. Supporting Factor Analysis

We also tested whether our a priori categorization was consistent with factor analyses on individual items at T1 and T2, respectively. We used maximum likelihood estimation with direct oblimin rotation, and redundant items measured at T2 (i.e., MERS/SARS, zombie movies and books) were not included in these factor analyses. Overall, we find good support for the four broad a priori categories we defined as separable categories.

From T1, items loaded on two factors accounting for 41% of the variance and their factor loadings largely confirmed our a priori categories. One factor captured Serious Diseases, with two items having loadings at least 0.50 and not cross-loading >0.30 on the second factor: the 1918 flu and SARS. The second factor captured Apocalyptic Events, with two items meeting the same criteria as above: zombie movies and zombie TV shows. HIV/AIDS, disease movies, and biblical depictions did not cleanly load on either factor. Consistent with our a priori intuitions, Normal Flu did not load on either factor, suggesting that respondents were treating it separately from the other items.

From T2, items loaded on two factors, accounting for 42.6% variance. One factor captured Major Upheavals, with five items having loadings of at least 0.50 and not also loading > 0.30 on the second factor: September 11, economic upheaval, social upheaval, natural disasters, and war. The second factor captured Serious Diseases, with Swine Flu and Ebola loading most strongly on this factor. Bubonic plague, as well as video and board games, did not cleanly load on either factor.

Importantly, these factor analyses broadly confirm our a priori category assignments with only slight differences. All analyses reported in the main text were conducted again using scales based on these factor analyses. Results were largely replicated; only 2 of the 24 possible effects of category similarity on behaviors became significant compared to analyses based on a priori categories. Once again, the overall pattern of results and the conclusions remain unchanged when the data are analyzed in this way.
